# Geographic and Genetic Population Differentiation of the Amazonian Chocolate Tree (*Theobroma cacao* L)

**DOI:** 10.1371/journal.pone.0003311

**Published:** 2008-10-01

**Authors:** Juan C. Motamayor, Philippe Lachenaud, Jay Wallace da Silva e Mota, Rey Loor, David N. Kuhn, J. Steven Brown, Raymond J. Schnell

**Affiliations:** 1 National Germplasm Repository, US Department of Agriculture, Agricultural Research Service, Subtropical Horticulture Research Station, Miami, Florida, United States of America; 2 MARS Inc., Hackettstown, New Jersey, United States of America; 3 CIRAD-Bios, UPR “Bioagresseurs de pérennes”, TA-A31/02, Montpellier, France; 4 CEPLAC/SUPOR (AMAZONIA), Belem-Pa, Brazil; 5 INIAP, Estación Experimental Pichilingue, Los Ríos, Ecuador; University of Chicago, United States of America

## Abstract

Numerous collecting expeditions of *Theobroma cacao* L. germplasm have been undertaken in Latin-America. However, most of this germplasm has not contributed to cacao improvement because its relationship to cultivated selections was poorly understood. Germplasm labeling errors have impeded breeding and confounded the interpretation of diversity analyses. To improve the understanding of the origin, classification, and population differentiation within the species, 1241 accessions covering a large geographic sampling were genotyped with 106 microsatellite markers. After discarding mislabeled samples, 10 genetic clusters, as opposed to the two genetic groups traditionally recognized within *T. cacao*, were found by applying Bayesian statistics. This leads us to propose a new classification of the cacao germplasm that will enhance its management. The results also provide new insights into the diversification of Amazon species in general, with the pattern of differentiation of the populations studied supporting the palaeoarches hypothesis of species diversification. The origin of the traditional cacao cultivars is also enlightened in this study.

## Introduction

Cacao is cultivated in the humid tropics and is a major source of currency for small farmers as well as the main cash crop of several West African countries. Its fruits (pods) contain the seeds (beans) that are later processed by the multi-billion-dollar chocolate industry. Average yields are about 300 kg per hectare but 3,000 kg/ha are often reported from field trials [1]. Genetic improvement of cacao through breeding has focused on increasing yield and disease resistance. To increase yield, breeders have capitalized on heterosis that occurs in crosses between trees from different genetic groups [2]. Traditionally, two main genetic groups, “Criollo” and “Forastero”, have been defined within cacao based on morphological traits and geographical origins [3]. A third group, “Trinitario”, has been recognized and consists of “Criollo”×“Forastero” hybrids [3]. In parallel, botanists described two subspecies: *cacao* and *sphaeorocarpum*, corresponding to “Criollo” and “Forastero” [4], [5], which, according to some authors, evolved in Central and South America, respectively [4], [5]. For other authors, “Criollo” and “Trinitario” should be considered as traditional cultivars rather than genetic groups [6] . Two other traditional cultivars have been described: Nacional and Amelonado [7]. Nonetheless, a sound classification of *Theobroma cacao* L. populations, based on genetic data, is lacking for the breeding and management of its genetic resources.

The Amazon basin contains some of the most biologically diverse tree communities ever encountered; tree species richness may attain three hundred species in one-hectare plots [8]. In cacao, flowers are hermaphrodites. However, it is an outcrossing species due to the action of self-incompatibility mechanisms in wild individuals, while the cultivated ones are generally self-compatible. Other Amazonian species of importance such as *Theobroma grandiflorum* show similar mating systems. Understanding the geographic pattern of differentiation of *T. cacao* would aid in implementing conservation strategies for many other species with similar mating systems and distribution within this important region.

Usually, population genetic studies require *a priori* classification of individuals into populations according to their geographical origin. Accessions of wild and cultivated cacao have been analyzed to study genetic relationships using passport data (available information on the origin of an accession) and molecular markers [9], [10]. However, using morphological data from the International Cocoa Germplasm Database (ICGD), it was estimated that misidentification of trees varies from 15 to 44% in germplasm collections [11]. Tree misidentification, or the substitution of one originally identified cacao genotype by another, occurs for various reasons (see Material and Methods below), including mislabeling of clones in the germplasm collection and on the germplasm collection maps, as well as replacement of grafted scions by the rootstock. Such tree misidentification makes it difficult to infer population structure. To understand population differentiation within *T. cacao* and to overcome the problem of mislabeled samples, we conducted a study using Bayesian statistics implemented through the software Structure [12]. The passport data from most of the individuals studied was not initially taken into account for the analyses to avoid a biased interpretation of the results due to the presence of mislabeled samples. This approach allowed us to obtain a structure of the genetic diversity within the species that contrasts sharply with the current knowledge in this area.

## Results and Discussion

One thousand two hundred forty-one individuals from different geographical origins and different collection trips were genotyped with 106 microsatellite markers. From the 106 markers used, data from 10 were excluded because of inconsistency across electrophoretic runs; a list of these microsatellites and their primer sequences is available in Table S1. Structure was used to infer the genetic structure; the model-based clustering algorithm implemented in Structure identifies clusters and subclusters by allelic frequencies. Individuals are placed in *K* clusters and can be members of multiple clusters, with membership coefficients summing to 1 across all *K* clusters. Putative *K* values are chosen in advance but can be modified across independent runs of the algorithm [12].

### Mislabeled accessions

Given the problem of sample mislabeling, several preliminary analyses to identify duplicated samples and preliminary runs with Structure were performed to exclude offtypes and human mediated hybrids. A total of 289 individuals were excluded from the final analyses (Table S2) and the remaining 952 individuals and their respective geographical origins are listed in Table S3. The number of excluded individuals reduced the sample size considerably for certain locations. The current restrictions for international access to wild germplasm from the Amazon basin have made it impossible to reconstitute the original sample sizes through new collection trips.

### Number of clusters

Searching for prudent genetic clusters, 10 Structure runs were performed for each *K* tested with *K* = 1 to *K* = 20. The highest number of *K* tested, 20, was an arbitrary and relatively low number given the high number of accessions and geographical locations sampled. However, the evolution across preliminary runs, with *K* = 1 to 20, of the proportion of individuals unequally assigned to the number of clusters studied, as well as the evolution of the posterior probability through the *K* tested, indicated that the range of *K* studied was suitable [13]. The number of clusters identified, using the approach explained in the Materials and Methods section (see below), was *K* = 10. For *K* = 10, 61.66% of the 952 retained individuals were identified under the same clustering scheme across the 10 runs performed, 33.93% under two schemes, 3.68% under three schemes and 0.73% under four schemes. The 10 clusters identified in the run with the highest estimated probability were named according to the geographical location or traditional cultivar most represented in that particular cluster: Marañon, Curaray, Criollo, Iquitos, Nanay, Contamana, Amelonado, Purús, Nacional and Guiana. This classification, which maintains the terms used to identify the traditional cultivars Amelonado, Criollo and Nacional, separates highly differentiated populations (see overall Fst value below) within what was previously classified as the Forastero genetic group.

Of the 952 individuals analyzed, 735 had a coefficient of membership equal to or higher than 0.70 to their respective identified cluster under the most probable clustering scheme and were retained to investigate the genetic substructure within the 10 clusters mentioned (see below). The 217 individuals with a coefficient of membership less than 0.70 were significantly (p<0.001) more heterozygous (51.6%) than the former ones (34.9%), even after excluding traditional cultivars [which are highly homozygous, [7]]. This suggests that they may have been collected in hybrid zones, i.e. areas where differentiated populations converge and hybrid offspring can arise. A strong differentiation was found for those 735 individuals grouped in the 10 clusters mentioned above. The overall Fst value (after 1000 bootstraps over the retained loci) was 0.46 (99% Confidence Interval: 0.44–0.49). Fst values over 0.25 are generally considered as indicators of significant population differentiation [14].

### Genetic clusters geographical distribution and subclustering

**Figure 1:**
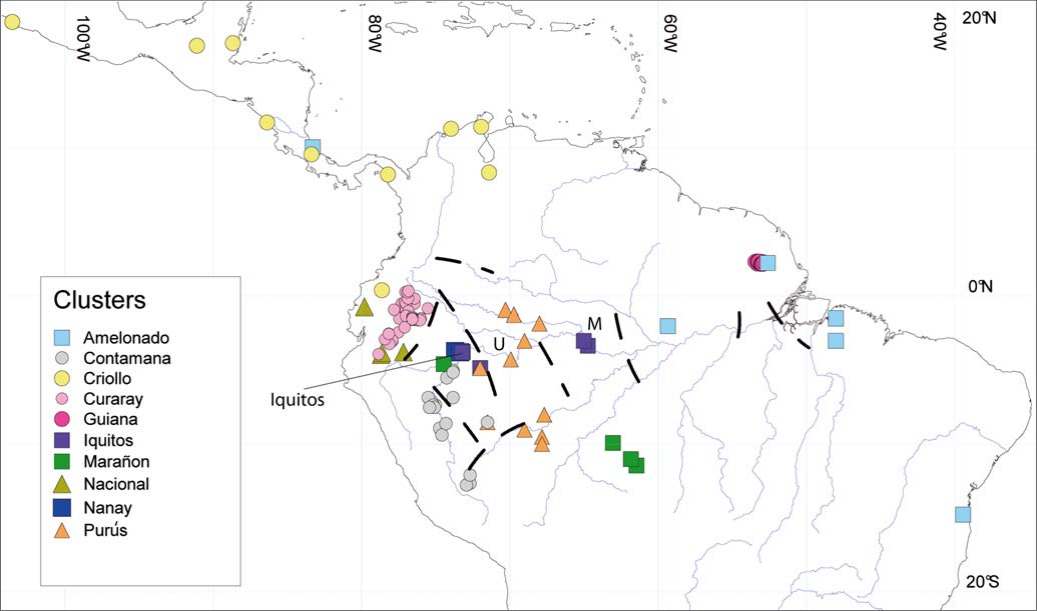
Localization of the origin of individuals analyzed; colors indicate the inferred genetic cluster to which they belong. Approximated location of Amazon ancient ridges (“palaeoarches”) is shown, after [26], in order of apparition clockwise: Fitzcarrald, Marañon, Serra do Moa, Iquitos, Vaupés, Carauari, Purús, Monte Alegre and Gurupa. U: Upper and M: Middle Solimðes.

Table S3 shows the highest coefficient of membership and the cluster name for the 735 individuals retained. Figure 1 displays the location where they were originally collected. The same symbol and color were used to display individuals, from a given location, that belonged to the same genetic cluster. Only individuals from the Criollo cluster are found in the Central American primary forests [Mexico [5] and Panama forests], while all ten clusters (including the Criollo one) are represented in the South American forests. Non-Criollo cacao types can be found in Central America (Figure 1, in Costa Rica, as the clone CC 267 or Matina 1–6) but only within existing farms, indicating that they were introduced more recently than the Criollo type. Nevertheless, what now appear as primary forests in Central America may have also been cultivated areas during pre-Columbian times. These data do not support the hypothesis that wild cacao evolved in Central America nor that simultaneous evolution of two subspecies, one in Central America and the other in the Amazon forest [5], occurred. The highest genetic diversity was found in the Upper Amazon region (see below), which is in agreement with the location of the putative center of origin of *T. cacao* L. [3]. The study of the genetic substructure in the subsample of 735 individuals indicated that the numbers of *K* identified in each of the 10 clusters (*K* = 3 to *K* = 5, with *K* = 1 to *K* = 15 tested), roughly corresponded to the number of geographical units and/or traditional cultivars found in each cluster. This correspondence was used to name each subcluster. From the 735 individuals belonging to the aforementioned 10 clusters, 559 had a coefficient of membership equal to or higher than 0.70 for one of the 36 subclusters grouping 5 or more individuals. These were retained for further analyses.

### Origin of traditional cultivars

The analysis of the genetic substructure within the clusters provides some clues to the origin of the Nacional cultivar. The Nacional cluster groups individuals from the Amazonian side of the Andes (Morona, Nangaritza and Zamora rivers, Table S3). However, these individuals are not included in the Nacional subcluster. This probably reflects centuries of human selection in the Ecuadorian Coast (Pacific side of the Andes). A strong resemblance of fruits of cacao trees from the Zamora River with those of the traditional cultivar Nacional has been reported [15]. In the case of the Amelonado traditional cultivar, our findings are less clear, as wild individuals from very distant locations as well as cultivated genotypes (Brazilian state of Bahia, Costa Rica and Ghana) are grouped in the Amelonado cluster. Nevertheless, within this cluster, we also find genotypes collected at the Para River. Historical data indicates that the Amelonado cultivar may have been domesticated from trees of this area.

### Neighbor joining tree

**Figure 2:**
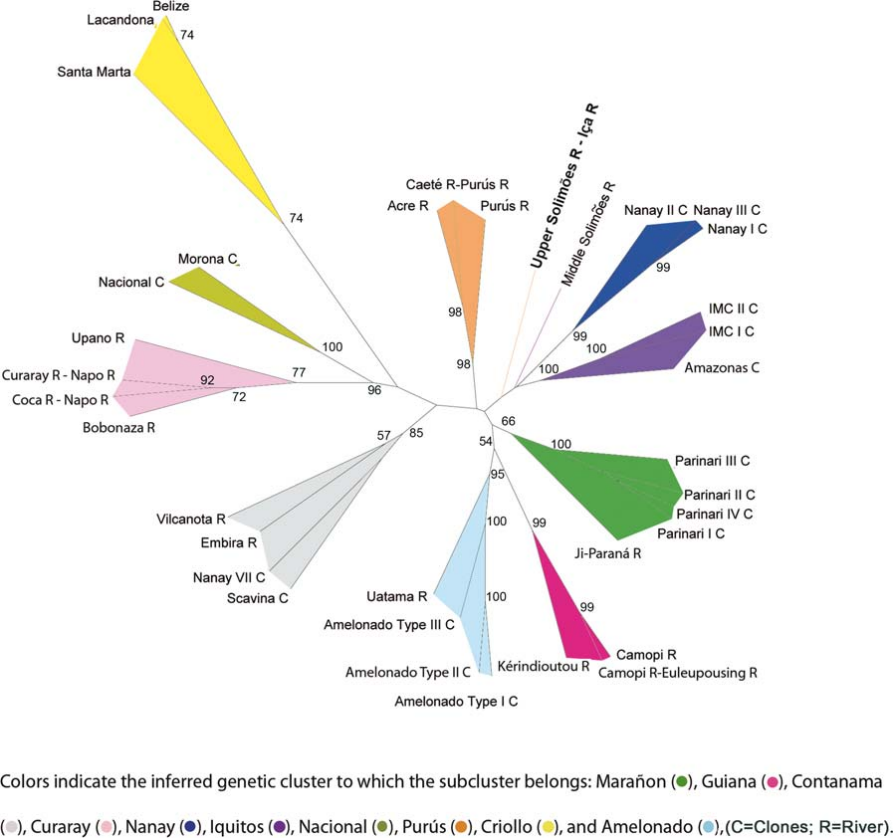
Neighbor joining tree from Cavalli-Sforza and Edwards genetic distance [16] matrix among the 36 subclusters identified using Structure (559 clones). Values represent percentages after bootstraps on the 96 loci retained.

Since more than one clustering scheme was found at *K* = 10, a complementary graphical cluster analysis was employed to visualize the relatedness among the subclusters identified. The Neighbor Joining method based on the Cavalli-Sforza and Edwards genetic distance [16] among the 36 subclusters (Figure 2) was used. The space between the branches of the subtrees is colored according to the major cluster to which each subcluster belongs (using the same codes as in Figure 1). In Figure 2, the 36 subclusters are grouped under the same clustering pattern observed using Structure, with the exception of the subcluster comprising individuals from the Upper Solimões and Iça River from the Purús cluster and the subcluster including individuals from the Middle Solimões from the Iquitos cluster. These subclusters did not group with other Structure clusters; rather they lie in-between their respective clusters and the next genetically closest cluster. This incongruity between the two clustering methods may be due to the fact that gene flow may occur throughout the Solimões River. Please note, in Table S3, that from the 559 individuals retained for this analysis, only individuals from the Upper Solimões and Iça River subcluster showed three clustering schemes across the 10 runs performed with *K* = 10 (all the others 1 or 2). The Upper and Middle Solimões are stretches of the Amazon River connecting the Upper Amazon (where other individuals from the Iquitos, Purús and Marañon clusters were collected) to its confluence with the Negro River. Comparing the number of migrants between all combinations of subclusters from different clusters indeed showed that the highest number of migrants was found between the Upper and Middle Solimões subclusters, from the Purus and Iquitos clusters respectively (Table S5). The second highest value was found between the Middle Solimões and Parinari IV from the Marañon cluster (Peruvian Upper Amazon). These results indicate that gene flow is extensive throughout the Amazon River making it difficult to cluster downstream introgressed populations.

### Putative family structure effect on the clustering pattern observed

Among the individuals analyzed, those collected by Pound [15] in the Upper Amazon [clone series : Nanay (NA), Iquitos Mixed (IMC), Parinari (PA), Scavina (SCA) and Morona (MO)(Table S3)] were in some cases derived from pods from common mother trees. At the time of his collection, rooting of cuttings or grafting were not reliable techniques and seed (beans) collection was the easiest method to collect germplasm from remote areas of difficult access. Although no specific information about the families that are descended from those trees is recorded by Pound, interpretation of Pound's collecting expedition report [17], suggest that pods from 14 to 17 trees were collected for the NA clone series, 2 trees for the IMC clone series, 7 to 20 trees for the PA clone series and 1 for the SCA clone series. Molecular data does not support the number of suggested trees at the origin of the Scavina clone series. Clones SCA-6 and SCA-12 have not a common haplotype, thus they are not derived from the same mother tree, which casts doubt on the number of trees at the origin of the other clone series.

Individuals from the Pound collection represent 90% of those from the Nanay cluster (NA series), 54% of the cluster Iquitos (IMC series), 68% of the cluster Marañon (PA series), 28% of the cluster Contamana (SCA series) and 27% of the cluster Nacional (MO series). The Pound collection due to its wide international distribution is the germplasm most commonly used in breeding programs worldwide.

To determine the effect of putative large families within the 10 clusters on the repeatability of the patterns identified, a subsample with 15 individuals from the 10 clusters (except for the Nanay cluster where 12 were selected) was studied using Structure. The number of individuals was reduced considerably in order to have a relatively balanced sample of genotypes per cluster with fewer individuals from the Pound collection. Simulations were performed following the same procedure described in Material and Methods and the best solution was estimated as *K* = 9, with individuals from the Nanay and Iquitos clone series forming one cluster and the other eight clusters composed of the same individuals as in the previous analysis (results not shown). However, this result may be the consequence of the small sample sizes studied, since ten differentiated clusters were observed on the larger sample size as indicated by Structure, Fst, Neighbor joining, number of alleles per subcluster and variance analyses (see below). Furthermore, individuals from the Nanay and Iquitos clone series were collected in a relatively small geographical area when compared to the origin of the other samples and, as seen in Figure 1, the locations of the Nanay and Iquitos samples overlap. Nonetheless, there are allele frequency differences that separate the Nanay and Iquitos clone series in two distinct clusters in the original sample size studied and for cacao geneticists and breeders these differences are important.

### Number of alleles per subcluster

A low and non-significant correlation (r = 0.23, p = 0.23) was observed between the number of alleles and the number of individuals of the 36 subclusters regardless of the great variation in population size. In spite of this fact, the rarefaction method [18], which standardizes the mean number of alleles per locus or the number of private alleles to the smallest number of individuals in a comparison, was employed to determine the number of private alleles per subgroup. Most subclusters contained private alleles. The number of private alleles across the 96 loci varied from 0.46 for the Nanay III subcluster to 25.89 for the Embira River subcluster (Table S4). The highest numbers of private alleles were found in subclusters from the Peruvian and Brazilian Upper Amazon region, close to the putative center of origin of the species [3].

### Genetic diversity variance analyses

Analyses of variance including the 36 subclusters were performed using 1,000 bootstraps on the individual genotypes at each hierarchical level with the software Arlequin [19]. All components of variance were highly significant (P<1×10^−5^). The analyses found 38.1% of the variance among the 10 clusters, 17.3% among subclusters within clusters and 44.6% within subclusters. When the same analysis was performed on wild and primitive material only, i.e. after excluding the traditional cultivars (Amelonado, Criollo and Nacional), the within subcluster variance increased to 50.4%. This value is similar to the within population variation values presented in other studies of cacao populations from the Amazon basin [20], [21]. It is important to emphasize that the degree of divergence among populations, as reflected by the percentage of variance among clusters, is much higher than previously reported [10], [20], [21] and may be the consequence of eliminating offtype individuals before performing population genetic analyses.

### Amazon diversification hypotheses

The high degree of differentiation among populations observed from the overall Fst value, number of private alleles and molecular variance analyses prompts questions about the mechanisms underlying such differentiation. At the highest hierarchical level (whole sample), our results do not support either the riverine or the refuge centers hypotheses of Amazon species diversification [22], [23], since populations belonging to the same cluster can be found across various major rivers/basins. The geographical distribution of the clusters does not correspond to the putative refuge centers proposed for other species in the region [24], [25]. Rather, the pattern of differentiation of the populations studied appears to be linked to potential dispersal barriers created by ancient ridges also called palaeoarches [see Figure 1, after [26]]. These palaeoarches, although they may involve different geological and geomorphological features and their distribution is difficult to locate precisely [27], seem to collocate with the boundaries of the cacao clusters distribution as represented in Figure 1. The ridges hypothesis has been evoked to explain the diversification pattern of other Amazonian species such as the dart-poison frog [*E. femoralis*, [28]] and piranha fish from the genera *Serrasalmus* and *Pygocentrus* [26]. These ancient ridges, no longer visible in the landscape, may have shaped the phylogeography of cacao and other Amazonian taxa by acting as ancient barriers to gene flow. Nonetheless, as mentioned above and seen in Figure 1, populations from several clusters are found in the same locality (e.g. Iquitos) without being bisected by any potential barrier (ridge, river). Concerning Iquitos, where several major rivers used for transportation converge, the presence of distinct populations could be due to ancient or modern human intervention. Further collection trips are needed to specifically investigate the association between the paleoarches and the genetic structure of *Theobroma cacao*. Most of the past collection expeditions have focused on collecting germplasm from individuals showing desired agronomic traits such as disease resistance [29]. Usually such collections were limited to only those few, or even unique individuals showing the desired agronomic advantages. This approach has thus reduced the number of samples available for analysis from a given location/population in diversity studies such as this one. Therefore, any subsequent collection trip should sample wild populations in sufficient numbers of individuals to be representative of the wild germplasm present, even if only leaves are collected from some of the trees. Through computer simulations of microsatellite data performed in our lab [30], little increase in the precision of gene diversity and F statistics estimates were found by increasing the sample size beyond 20 trees per population. Twenty would therefore be an ideal number of individuals to sample per location/population.

### Conclusion

The results presented here lead us to propose a new classification of cacao germplasm into 10 major clusters, or groups: Marañon, Curaray, Criollo, Iquitos, Nanay, Contamana, Amelonado, Purús, Nacional and Guiana. This new classification reflects more accurately the genetic diversity now available for breeders, rather than the traditional classification as Criollo, Forastero or Trinitario. We encourage the establishment of new mating schemes in the search of heterotic combinations based on the high degree of population differentiation reported. Furthermore, we propose that germplasm curators and geneticists should use this new classification in their endeavor to conserve, manage and exploit the cacao genetic resources.

## Materials and Methods

### DNA Extraction

Leaf material was collected from the germplasm listed in Table S2 and S3. DNA extraction was performed on 200 mg samples of leaf tissue using the FastDNA kit (QBIOgene, Carlsbad, CA) and a FastPrep FP 120 Cell Disrupter (Savant Instruments, Inc.; Holbrook, N.Y.). The kit protocol for plant tissue was followed, including the optional SPIN protocol. Tissue was homogenized using the Garnet Matrix and two ¼ inch spheres as the Lysing Matrix combination, at speed 5 for 30 s, repeated three times. DNA was quantified on a DynaQuant 200 Spectrophotometer (Amersham Pharmacia; Piscataway, CA). All samples were diluted 1∶20 to obtain a concentration of ∼2.5 ng·µL^−1^.

### Microsatellite markers and Polymerase Chain Reaction (PCR) amplification

The microsatellite markers used in this study were previously reported [31]–[33]. The marker names, primer sequences, annealing temperatures, size and dye utilized are listed in Table S1. PCR amplifications were accomplished using the protocol previously reported in [34]. PCR amplification reactions were carried out in a total volume of 10 µL, containing 2.5 ng·µL^−1^ genomic DNA, and multiplex reactions were carried out in a total volume of 25 µl with 6.25 ng·µL^−1^ genomic DNA. All PCR reactions contained 0.05 U·µL^−1^ Amplitaq (Applied Biosystems, Inc.; Foster City, CA), 0.2 mM dNTPs, 0.4 µM each forward and reverse primers, 2 mg·ml^−1^ BSA and 1× GeneAmp PCR buffer [1.5 mM MgCl_2_, 10 mM Tris-HCl pH8.3, 50 mM KCl, 0.001% (w/v) gelatin]. Thermal cycling profile consisted of: 4 min denaturation at 94°C; followed by 33 cycles of denaturation at 94°C for 30 s, 1 min at appropriate annealing temperature for each primer and 1 min extension at 72°C ; and a final 5 min 72°C extension.

For multiplex primer reactions the extension temperature was changed to 65°C and final extension increased to 7 min. PCR was carried out on a DNA Engine tetrad thermal cycler (M J Research, Inc.; Watertown, MA.). Multiplex primer reactions were performed for combinations of primers with matching annealing temperatures but differing size ranges and dye labels.

### Electrophoresis

Capillary electrophoresis (CE) was performed on an ABI Prism 3100 Genetic Analyzer (Applied Biosystems, Inc) using Performance Optimized Polymer 4 (POP 4; Applied Biosystems, Inc), or an ABI Prism 3730 Genetic Analyzer (Applied Biosystems, Inc.) using Performance Optimized Polymer 7 (POP 7; Applied Biosystems, Inc.). Samples were prepared immediately prior to electrophoresis by adding 1 µL of PCR product to 20 µL of deionized water and 0.1 µL of GeneScan 500 ROX or GeneScan ROX 400HD size standard (Applied Biosystems, Inc.), then denatured at 95°C for 30 s, and chilled on ice. The default run module for microsatellite analysis was used. Resulting data were analyzed with GeneMapper 3.0 (Applied Biosystems, Inc.) for internal standard and fragment size determination and for allelic designations. The same size standard was used on all samples analyzed for each marker.

### Germplasm

A fingerprint database for the 96 retained microsatellite loci is available for all individuals (1236) with fewer than 5% (average for the database 2.7%) missing data at http://www.ars.usda.gov/Research/docs.htm?docid=16432.

The 952 individuals retained after the removal of the off-types are clones, i.e. vegetatively propagated genotypes. These genotypes were collected as budwood or seeds during various collecting trips, or were selected from cultivated material (“cultivars”), and originate from 12 countries. Wild or primitive (i.e. cultivated on a small scale, and whose origin is local or geographically rather close) germplasm has been collected from 1937 to 2005, in numerous collecting trips in Brazil, Colombia, Ecuador, Peru, French Guiana and Central America [see [15] for a review, and [35]]. A great part of the wild or primitive material studied originates from Peru (416 clones, 44% of the total), where the species' putative center of origin is located. Other major contributions are from Brazil (248 clones, 26%) and Ecuador (172 clones, 18%), but these also include cultivated clones. These cultivated clones belong to the traditional cultivars named Criollo, Amelonado and Nacional, whose wild origins are poorly known. Criollos from Central America, true “West African Amelonado” from Ghana, a Matina clone from Costa Rica, several Común clones from Brazil and Nacional clones from the Pacific coast of Ecuador were included to study their relatedness to wild or primitive genotypes. Some Amelonado selections (for example, EEG and SIC clone series from Brazil) were also included, but all Trinitario and Trinitario×Amazonians clones, representing human created hybrids between traditional groups Criollo and Amelonado and Trinitario×wild genotypes from the Amazon basin were excluded. Table S3 lists the 952 clones, with their name, lab sample id, passport data (approximate longitude and latitude of collection, country of origin, cluster and subcluster to which they belong with their respective coefficient of membership). Table S3 was established after the International Cocoa Germplasm Database [35], and after [6], [11], [29], [36]–[40].

### Offtype detection

To reduce noise caused by the excessive number of mislabeled genotypes, preliminary analyses were performed to identify mislabeled clones. First, identical genotypes were identified using the software Cervus 2.0 [41]. One hundred pairs of identical genotypes were identified. Two samples were considered identical if they shared at least 95% of their alleles for the 96 microsatellites retained. Since we detected up to 5% of genotyping errors (average 1.85%) in some cases when repeated samples were compared, a 95% criterion for the proportion of shared alleles was employed. However, within certain clusters with narrow genetic diversity such as Nanay or Criollo, even individuals collected in different locations and propagated by seed, were identical for those 96 microsatellites, therefore indistinguishable. In clusters of individuals so highly homozygous and showing such low diversity, only reliable passport data can be used to discern if two individuals are identical or not. When two identical genotypes were found with different names, we retained the one that clustered with the clones from the expected location after preliminary runs with Structure. Some samples from the clones studied were duplicated because their field positions in the germplasm collections were unclear. When their fingerprints were identical, one was discarded. Otherwise, only the correctly clustered sample according to the procedure described above was retained. In some cases, two individuals with the same identification and different fingerprints were received, and clustered in the same group. This can be explained because for certain collection trips [36], seedlings from the same original mother tree were not independently identified and were labeled with the name of the mother tree. In these cases, we kept the same name but we have a lab ID (TC number), which allows us to trace its location to the field position of the respective germplasm collection. Samples from Pound's collection trips [29] were collected from the Cocoa Research Unit Marper station (Island of Trinidad) in 2003 and compared to samples previously collected from the same trees in 2001 [20]. Between 2001 and 2003, the original scion for many accessions at Marper died (the rootstock remained). Fingerprints using 12 microsatellites from the 2001 and 2003 samples were compared to identify non-matching samples (due to potential DNA sampling from the remaining rootstock). DNA samples from the 2001 collection were not available in sufficient quantity for fingerprinting with 96 loci, so when the fingerprints didn't match, the nonmatching 2003 samples were discarded. For most germplasm collections, seedlings from the traditional cultivars are used as rootstocks. These are grafted with scions from the germplasm collection expeditions. To identify rootstock genotypes that have overgrown the scions, resulting in misidentification errors, we used clones known to belong to the Nacional, Amelonado, Trinitario and Criollo traditional cultivars [6]. These were set as reference genotypes using the population flag command of Structure [13]. This command uses a clustering model algorithm that incorporates prior population information about the genotypes to cluster. The algorithm uses priors for each individual to calculate probabilities that it is a migrant for the particular *K* tested or that it has a migrant ancestor [13]. Fifty samples were identified as rootstock this way. Trinitario clones included as reference genotypes, as well as other human mediated hybrids, were not included in the final analyses. Retained individuals after all these procedures were assigned to their respective cluster, according to the *K* = 10 run with the maximum likelihood (see below Cluster analyses), if they had a coefficient of membership to that cluster equal or higher than 0.70. Some cases of mislabeling were detected through identification of individuals clustering with a high coefficient of membership (over 0.70) in a different cluster from most of the individuals collected from the same expected locality (according to passport data). In these cases the individuals were excluded from analyses. For the Nanay clones, which are generally considered as a primitive population from one locality and without detailed passport information, several individuals dispersed in several clusters. In this case, they were retained since, according to historical records, several populations were collected in different localities although all individuals were identified under the name Nanay or Pound [15]. For some samples it was not possible to obtain any passport data and perform any of the comparisons mentioned above and thus they were discarded.

### Cluster analyses

For the preliminary runs, the correlated allele frequencies model of Structure [12] was used with runs of 20,000 iterations after a burn-in of length 10,000. To accurately determine *K*, 10 runs of 200,000 iterations after a burn-in period of length 100,000 were performed for each *K* tested after excluding all the offtype individuals. We chose the *K* that maximized the posterior probability of the data, accounting for the evolution of the plot with the posterior probability across runs for different *K* values. For increasing numbers of *K* tested, when numerous genetic groups exist within the data, the posterior probability will increase until it reaches a relative plateau. We chose the *K* value at the beginning of each of these relative plateaus; the *K* value that makes salient a prominent change of the posterior probability slope. This approach is equivalent to taking the second order rate of change of the likelihood function with respect to *K* (Δk) [42]. When complex datasets that include many genetic groups are analyzed with Structure, the algorithm converges to numerous solutions for a given number of *K* or cluster [12]. In these cases, estimated probabilities differ for the same number of assumed clusters *K* tested. The clustering scheme selected for *K* = 10 was that from the run for *K* = 10 with the highest estimated probability. The same procedures described were also employed to identify the most probable *K* within the 10 clusters.

### F statistics

Microsatellite data from the 735 individuals with coefficient of membership equal or higher than 0.70 for the ten clusters identified with Structure was used to calculate the overall Fst value. One thousand bootstraps were performed over loci to calculate the 99% confidence interval using the software Fstat [43].

### Plotting of individuals on Central and South American map

Using the coordinates from the passport data of the individuals from the 10 clusters identified, their position was plotted on a map of Central and South America [created with Arcgis 9 [44], using the GIS software MapInfo Professional 8.5 [45] Each genotype was labeled according to the cluster to which it belongs as identified using Structure.

### Estimation of the number of private alleles in subclusters

The rarefaction approach was implemented using the software HP-Rare 1.0 [18]. The number of alleles that would occur in smaller samples of individuals is estimated, based on the frequency of distribution of alleles at a locus. Rarefaction is typically used to standardize the mean number of alleles per locus or the number of private alleles to the smallest N in a comparison [18]. Since the smallest subcluster was composed of five individuals, the standardization was performed through sampling 5 gene copies.

### Variance analysis

Using the software Arlequin 3.01 [19] the genetic structure of the sample was analyzed by analysis of variance. The hierarchical analysis of variance, partitions the total variance into covariance components due to intra- and inter- level gene frequency differences. The covariance components are used to calculate fixation indices. The significance of the fixation indices is tested using a non-parametric permutation approach consisting in permuting individual genotypes at intra and inter hierarchic levels.

### Neighbor joining tree of subclusters

The Cavalli-Sforza and Edwards genetic distance [16] was calculated among the 36 subclusters identified (with Structure) and utilized to construct a neighbor joining tree after 10,000 bootstrap estimations on the 96 loci retained using Populations [46]. The unrooted tree was plotted and edited using the software Treedyn [47].

### Gene flow estimation

Gene flow among the 36 subclusters was estimated using Popgene Version 1.3.2 [48] by calculating the number of migrants (Nm) based on F statistics [49].

## Supporting Information

Table S1 Detailed list of microsatellites primer sequences.
(0.04 MB XLS)

Table S2 Detailed list of the 289 individuals that were excluded from the final analyses and reasons for exclusion.
(0.07 MB XLS)

Table S3 List of the 952 retained clones.
(0.77 MB XLS)

Table S4 Cumulated number of private alleles within the 36 subclusters identified for the 96 loci studied.
(0.06 MB DOC)

Table S5 Estimates of gene flow (Nm) among subclusters based on F statistics.
(0.04 MB XLS)
