# Supplementary material for: Geographic and Genetic Population Differentiation of the Amazonian Chocolate Tree (Theobroma cacao L)
Source: PLoS One. 2008 Oct 1;3(10):e3311. doi: 10.1371/journal.pone.0003311 (PMC2551746; doi:10.1371/journal.pone.0003311)
Supplement: Table S4 — Cumulated number of private alleles within the 36 subclusters identified for the 96 loci studied. (0.06 MB DOC) [file pone.0003311.s004.doc]

| Subcluster | **N** | **Cumulated number of private alleles** | **Cluster** | **Subcluster** | **N** | **Cumulated number of private alleles** | **Cluster** |
| --- | --- | --- | --- | --- | --- | --- | --- |
| Parinari III C | 11 | 4.52 | Marañon | Nanay I C | 33 | 0.59 | Nanay |
| Parinari II C | 7 | 2.96 | Marañon | Middle Solimões R | 7 | 6.99 | Iquitos |
| Parinari IV C | 27 | 2.71 | Marañon | IMC II C | 13 | 1.89 | Iquitos |
| Ji-Paraná R | 35 | 14.47 | Marañon | Amazonas C | 7 | 7.27 | Iquitos |
| Parinari I C | 22 | 1.29 | Marañon | IMC I C | 43 | 1.99 | Iquitos |
| Camopi R | 10 | 0.92 | Guiana | Nacional | 20 | 4.86 | Nacional |
| Camopi-  Euleupousing R | 21 | 0.84 | Guiana | Morona C | 9 | 9.85 | Nacional |
| Kérindioutou R | 10 | 0.87 | Guiana | Acre R | 24 | 10.93 | Purús |
| Scavina C | 26 | 25.75 | Contamana | Upper Solimões R - Iça R | 11 | 20.02 | Purús |
| Embira River | 6 | 25.89 | Contamana | Purús R | 6 | 9.35 | Purús |
| Vilcanota R | 7 | 17.66 | Contamana | Caeté R - Purús R | 21 | 9.67 | Purús |
| Nanay VII C | 9 | 5.28 | Contamana | Santa Marta | 7 | 1.91 | Criollo |
| Bobonaza R | 5 | 7.13 | Curaray | Lacandona | 14 | 2.28 | Criollo |
| Upano R | 13 | 24.47 | Curaray | Belize | 6 | 1.82 | Criollo |
| Coca R-Napo R | 33 | 3.73 | Curaray | Amelonado Type I C | 17 | 2.03 | Amelonado |
| Curaray R-Napo R | 14 | 3.75 | Curaray | Uatuma R | 8 | 5.34 | Amelonado |
| Nanay II C | 8 | 0.52 | Nanay | Amelonado Type III C | 5 | 4.11 | Amelonado |
| Nanay III C | 21 | 0.46 | Nanay | Amelonado Type II C | 23 | 0.88 | Amelonado |
